# Far from the threatening crowd: Generalisation of conditioned threat expectancy and fear in COVID-19 lockdown

**DOI:** 10.3758/s13420-024-00625-4

**Published:** 2024-01-29

**Authors:** Simon Dymond, Gemma Cameron, Daniel V. Zuj, Martyn Quigley

**Affiliations:** 1 School of Psychology, Swansea University, Singleton Campus, Swansea, SA2 8PP UK; 2 Department of Psychology, Reykjavík University, Menntavegur 1, Nauthólsvík, 101 Reykjavík, Iceland; 3 School of Psychological Sciences, University of Tasmania, Locked Bag 1342, Launceston, TAS 7250 Australia

**Keywords:** Generalisation, Threat expectancy, Fear conditioning, COVID-19, Worry

## Abstract

Fear and anxiety are rarely confined to specific stimuli or situations. In fear generalisation, there is a spread of fear responses elicited by physically dissimilar generalisation stimuli (GS) along a continuum between danger and safety. The current study investigated fear generalisation with a novel online task using COVID-19-relevant stimuli (i.e., busy or quiet shopping street/mall scenes) during pandemic lockdown restrictions in the United Kingdom. Participants (*N* = 50) first completed clinically relevant trait measures before commencing a habituation phase, where two conditioned stimuli (CSs; i.e., a busy or quiet high street/mall scene) were presented. Participants then underwent fear conditioning where one conditioned stimulus (CS+) was followed by an aversive unconditioned stimulus (US; a loud female scream accompanied by a facial photograph of a female displaying a fearful emotion) and another (CS-) was not. In a test phase, six generalisation stimuli were presented where the US was withheld, and participants provided threat expectancy and fear ratings for all stimuli. Following successful conditioning, fear generalization was observed for both threat expectancy and fear ratings. Trait worry partially predicted generalised threat expectancy and COVID-19 fear strongly predicted generalised fear. In conclusion, a generalisation gradient was evident using an online remote generalisation task with images of busy/quiet streets during the pandemic. Worry and fear of COVID-19 predicted fear generalisation.

**Supplementary Information:**

The online version contains supplementary material available at 10.3758/s13420-024-00625-4.

## Introduction

The coronavirus disease 2019 (COVID-19) pandemic resulted in significant loss of life, with the United Kingdom (UK) one of the worst affected countries (Dong et al., 2020). To prevent transmission of the disease, the UK government introduced national ‘lockdown’ periods involving school closures, restricting contact with other household members, and banning all non-essential travel. These lockdowns had a detrimental impact on mental health (Carr et al., 2021; Chandola et al., 2020), with high levels of anxiety related to COVID-19 and notable impacts on well-being (Office for National Statistics, 2020).

During lockdown, public reminders of the threat posed by COVID-19 transmission could be found in images of busy shopping streets, crowds at sporting events, and other situations where social distancing was either difficult or impossible. The heightened threat value of such previously benign images of crowds may have been most acute during lockdown when the population were instructed to stay at home and prevented from in-person socialising. As a result, it is likely that pandemic restrictions and the contrast-effect of witnessing reminders of activities now forbidden may have triggered sustained levels of anxiety, fear, and avoidance (Ford et al., 2021; Renard, 2016; Vander Veer et al., 2012). Despite this, little is known from experimental tasks conducted remotely on the impact of *current* living in lockdown circumstances on fear/threat learning.

In the current study, we examined whether a fear conditioning and generalisation paradigm aids in the investigation of perceived threat value evoked by street scenes varying from quiet to busy during a period of national COVID-19 lockdown restrictions. Fear conditioning is a widely used transdiagnostic paradigm for investigating pathogenic markers of fear in anxiety and stressor-related disorders (Craske et al., 2022; Lonsdorf et al., 2017; Zuj et al., 2016). During the acquisition or conditioning of fear, a neutral stimulus comes to predict an aversive unconditioned stimulus (US; e.g., electric shock) and is then referred to as a conditioned stimulus (CS+), while another stimulus (i.e., CS-) comes to predict the absence of the US. Once differential conditioned fear is established, generalisation tests may be conducted with presentations of stimuli perceptually or conceptually similar to the CSs, in the absence of the US (Dymond et al., 2015). Fear generalisation is usually evident from a gradient-like range of responses elicited by generalisation stimuli (GS) along the intermediate range between danger (CS+) and safety (CS-) stimuli (Beckers et al., 2023; Cooper et al., 2022; Dymond et al., 2015; Fraunfelter et al., 2022).

For instance, Lissek et al. (2008) demonstrated fear gradients of the eye-blink startle reflex and risk ratings to visually presented rings that varied in size from a ring paired with shock (CS+). During acquisition, electric shock followed the CS+ ring on nine of the 12 trials (75% reinforcement schedule), while the CS- was never followed by shock (rings serving as CS+ and CS- were counterbalanced across participants). To test whether conditioned fear generalised from the CS+ to other stimuli, eight different unreinforced rings (i.e., presented in the absence of the US), ranging in size between the CS+ and CS-, were used as GSs. During the generalisation test, fear responses were observed for stimuli that visually approximated the CS+ and gradually decreased as the GSs became more dissimilar from the CS+.

To date, conditioned fear generalisation has rarely been investigated with salient visual stimuli or without aversive electric shock as the US (Dymond et al., 2015). Notable exceptions, however, include studies of the generalisation of social learning in the context of aesthetics (Boddez et al., 2019) and trust (FeldmanHall et al., 2018). The fear generalisation model has also been extended to neurobehavioural studies employing facial features (Haddad et al., 2012, 2013), conceptual categories (e.g., Marstaller et al., 2021; Morey et al., 2020), and visuospatial attention tasks (Dowd et al., 2016), among others. It is therefore clear that the fear generalisation paradigm has enormous potential in explaining the spread of learning across relevant related situations. This potential is extended still further with incorporation of remote (i.e., online and smartphone-based) delivery of the experimental task with a range of new populations (e.g., Alcalá et al., 2024; Hauck et al., 2022; McGregor et al., 2021). For instance, Purves et al. (2019) and McGregor et al. (2021) developed and validated a smartphone app for the study of fear conditioning and extinction, while Berg et al. (2022) recently conducted a feasibility study of an online-delivered US calibration procedure and PowerPoint presentation-based fear-conditioning paradigm. To date, however, no study has investigated both online fear conditioning and generalisation with a suitably calibrated and validated programmed task.

In the present study, we deployed an online fear conditioning and generalisation task based on our previous work (Cameron et al., 2022, 2023) for remote administration while COVID-19 national lockdown restrictions were in place. Using images of busy or quiet shopping street/mall scenes (i.e., images of activities the population were at the time prevented from doing), we paired counterbalanced shopping scenes with an unpleasant aversive US and then presented several intermediate, unreinforced scenes. We recorded trial-by-trial ratings of the likelihood of the US occurring (i.e., threat expectancy) and ratings of how afraid participants were of the CSs and GSs at the end of each block of trials (i.e., fear ratings). We predicted that a generalisation gradient would be evident in threat expectancy and fear ratings elicited by conditioned and generalised cues in an online task incorporating an auditory aversive US. We also sought to examine potential associations between generalisation performance and clinically relevant personality factors such as fear of COVID-19, worry, depression, anxiety, and intolerance of uncertainty (Arnaudova et al., 2017; Bauer et al., 2020; Cooper et al., 2022; Dunning & Hajcak, 2015; Sep et al., 2019; Wurst et al., 2021). Given the somewhat heterogeneous findings on the role of clinically relevant measures in predicting fear generalisation (Arnaudova et al., 2017; Dunning & Hajcak, 2015; Sep et al., 2019), we undertook exploratory analysis of these factors in predicting threat and fear responses within the present sample.

## Method

### Participants

Participants were recruited via Prolific Academic, an online recruitment platform. Inclusion criteria consisted of being aged 18 years or older, currently residing in the UK, not pregnant, and with no reported neurological, hearing, or vision difficulties. A total of 57 respondents initiated the study; one (1.7%) withdrew during the demographics data collection phase, five (8.7%) did not progress beyond the sound check stage, and one (1.7%) left during *threat conditioning*. The final sample consisted of 50 participants (all female, M_age_ = 31.54 years, SD = 10.87 years) and data collection was completed in July 2021 prior to a change in national COVID restrictions. A sensitivity analysis was conducted using G*Power v3.1 (Faul et al., 2007), which showed that with ⍺ = 0.05, Power (1 – β) = 0.80, five predictors, and a single regression coefficient, our regression analyses should be sensitive to detect an effect size of Cohen’s *f*^2=^ .16 or an *R*^2^ of .14. Ethical approval was provided from Swansea University’s School of Psychology Ethics Committee

### Stimuli

Conditioned and generalised stimuli consisted of colour images of a street scene (of Queen Street, Cardiff, Wales, image taken by The Western Mail newspaper in winter 2020) that varied from quiet to busy according to the number of people present. The chosen stimuli were deemed to be sufficiently salient and representative of the prevailing social-public health conditions in the UK at the time, to be equally likely to elicit pre-experimental appetitive and non-appetitive functions, and to illustrate the then-novel presence of face coverings worn by people engaging in otherwise familiar activities (i.e., walking on a popular shopping street). Beginning with the original, busy street scene, individual scenes were edited using photo-editing software; subsequent images decreased by the presence of six people per GS.

**Fig. 1:**
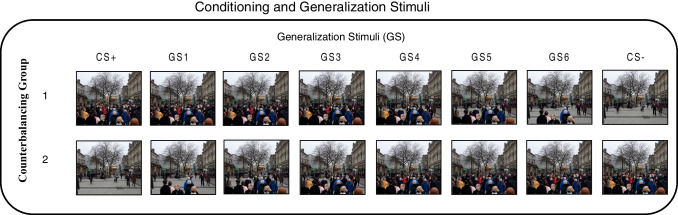
The street scenes which served as the conditioning and generalisation stimuli throughout the task for both counterbalanced groups

A total of eight street scenes were created (see Fig. 1), two of which (i.e., the quietest and busiest scenes) were designed as the CS+ and CS-, respectively (counterbalanced across participants), while the remaining six intermediary scenes were designated generalisation stimuli (GS1-6).

The US was a compound visual-auditory stimulus consisting of a facial photograph of a female displaying a fearful emotion from the NimStim set of facial expressions (model number: “03F_FE_O”) paired with a 2-s shrieking scream of approximately 90 dB (Cameron et al., 2022; Neumann & Waters, 2006; Tottenham et al., 2009).

### Trait measures

Participants completed the 16-item *Fear of Coronavirus Questionnaire* (FCQ; Mertens et al., 2020, 2021; α = .86), the *Penn State Worry Questionnaire* (PSWQ; Meyer et al., 1990; α = .93), *Patient Health Questionnaire* (PHQ – 9; Kroenke et al., 2001; α = .90), *Generalised Anxiety Disorder* assessment (GAD-7; Spitzer et al., 2006; α = .93), and the *Intolerance of Uncertainty Scale (IUS) – short form* (Carleton et al., 2007; α = .92). Sum scores were calculated for each of these measures.

### Procedure

The experiment was built and hosted online using Gorilla Experiment Builder (www.gorilla.sc; Anwyl-Irvine et al., 2020) and data collection occurred on 12 July 2021. First, participants completed the questionnaires before commencing a US-calibration (‘sound check’) to ensure they could hear the US. They were then instructed that on each trial one of two street scenes will be followed by the loud scream and to rate their expectancy of the US using the computer mouse on a sliding scale ranging from 0 (“*highly unlikely a scream*”) to 100 (“*highly likely a scream*”). CS duration was 8 s; the threat expectancy scale appeared below the CS 3 s after trial onset and remained onscreen for 5 s. The US was presented for 2 s at CS+ offset. No US was presented on any CS- trial. Each trial was followed by an inter-trial interval (ITI) of a white screen for 3 s and a black fixation cross for 250 ms. At the end of each phase, each CS was presented, and participants rated how afraid they were of the images. The fear rating scale ranged from 0 (“*unafraid*”) to 10 (“*afraid*”).

Stimulus presentation was pseudo-randomised throughout, with no more than two consecutive trials of each CS. All participants took part in three phases: *habituation*, *fear conditioning*, and *generalisation test* (Fig. 1). During *habituation*, the CS+ and CS- were each presented once in the centre of the screen in the absence of the US. In *fear conditioning*, the CS+ and CS- trials were each presented six times (12 trials in total). The US was presented immediately upon CS+ offset on four of six trials (i.e., a 67% CS-US reinforcement schedule), and never following the CS-. During the *generalisation test* phase, all conditioned and generalised stimuli were presented four times (32 trials in total). A steady-state generalisation test format was conducted (i.e., with partially reinforced presentations of CS+ and non-reinforced presentations of GSs; Honig & Urcuioli, 1981) in which the US was presented following two of four CS+ trials (i.e., a 50% CS-US reinforcement schedule), while all US deliveries were withheld following CS- and GS trials. Following this phase, participants were debriefed and compensated.

### Data analysis

Analyses were conducted in JASP (version 0.14.1; Love et al., 2015) with ⍺ = .05. The dataset can be found on the Open Science Framework website (Here). Separate analyses were performed for threat expectancy and fear ratings across phases collapsed across counterbalanced groups. Further sub-group analysis of each counterbalanced group for each conditioning phase is provided in the Online Supplementary Materials (OSM).^1^ Ratings provided during habituation were analysed using paired-samples *t*-tests. Threat expectancy ratings provided during threat conditioning were analysed using a repeated-measures analysis of variance (ANOVA) that compared stimulus type (CS+, CS-) and trial (T1-6) as within-subjects variables, whilst a paired-samples *t*-test was performed on the fear ratings. Threat expectancy and fear ratings during generalisation testing were examined using a repeated-measures ANOVA with the factor stimulus type (CS+, GS1-6, CS-). A polynomial linear contrast analysis was also performed to examine the pattern of threat expectancy and fear ratings during this phase. Greenhouse–Geisser-corrected *F*-ratios and degrees of freedom are reported where the assumption of sphericity was not met, and Bonferroni corrections were applied to all planned and post hoc comparisons for all measures within each phase consistently (e.g., Cameron et al., 2022, 2023).

Bayesian analyses were also undertaken, when null effects were observed, using JASP’s default priors to estimate the Bayes Factor_10_ (BF_10_; Krypotos et al., 2017; Rouder et al., 2012). As such, a Cauchy prior centred around 0 with a width of .707 was used for Bayesian paired-samples t-tests, whilst a multivariate Cauchy prior (also centred on 0) with a fixed effects factor of r = .5 and a random-effects of r = 1 was used for Bayesian ANOVAs. For Bayesian regressions the Jefferys-Zellner-Siow (JZS) prior with an r scale of covariates of ,354 (Rouder & Morey, 2012) was used. BF_10_ values > 1 and < 1 represent the likelihood of the data under the alternative hypothesis or the null hypothesis. For interaction effects, Bayes factors were calculated by comparing the model with the interaction against the model with the two main effects but without the interaction.

To examine whether scores on the questionnaires predicted threat expectancy and fear ratings to the generalisation stimuli, multiple regressions were performed. Predictor variables included each of the questionnaire measures (FCQ, PSWQ, PHQ-9, GAD-7, IUS). Multicollinearity of variables was examined by inspection of the variance inflation factors (VIF) and tolerance; both the average VIF and tolerance were < 2 and > .2, respectively indicating an unbiased model. Average threat expectancy and fear ratings across generalisation stimuli (i.e., GS1-GS6) served as the outcomes to ensure each of the GS stimuli were weighted equally (we acknowledge, however, that there are other valid indexing measures of generalisation e.g., Lenaert et al., 2016; Mertens et al., 2021).

## Results

### Habituation

As predicted, threat expectancy ratings across habituation were comparable for CS+ (*M* = 26.92; SD = 28.55) and CS- (*M* = 21.94; SD = 17.38), *t* (14) = -.52, *p* = .61, *d* = .13, 95% CI [-.64, .38], BF_10_ = .30.

Fear ratings were also comparable for both stimuli (CS+: *M* = 2.31, *SD* = 2.44; CS-:* M* = 2.75, *SD* = 2.95), *t* (39) = -1.12, *p* = .27, *d* = -.18, 95% CI [-.49, .14], BF_10_ = .31.

### Fear conditioning

**Fig. 2.**
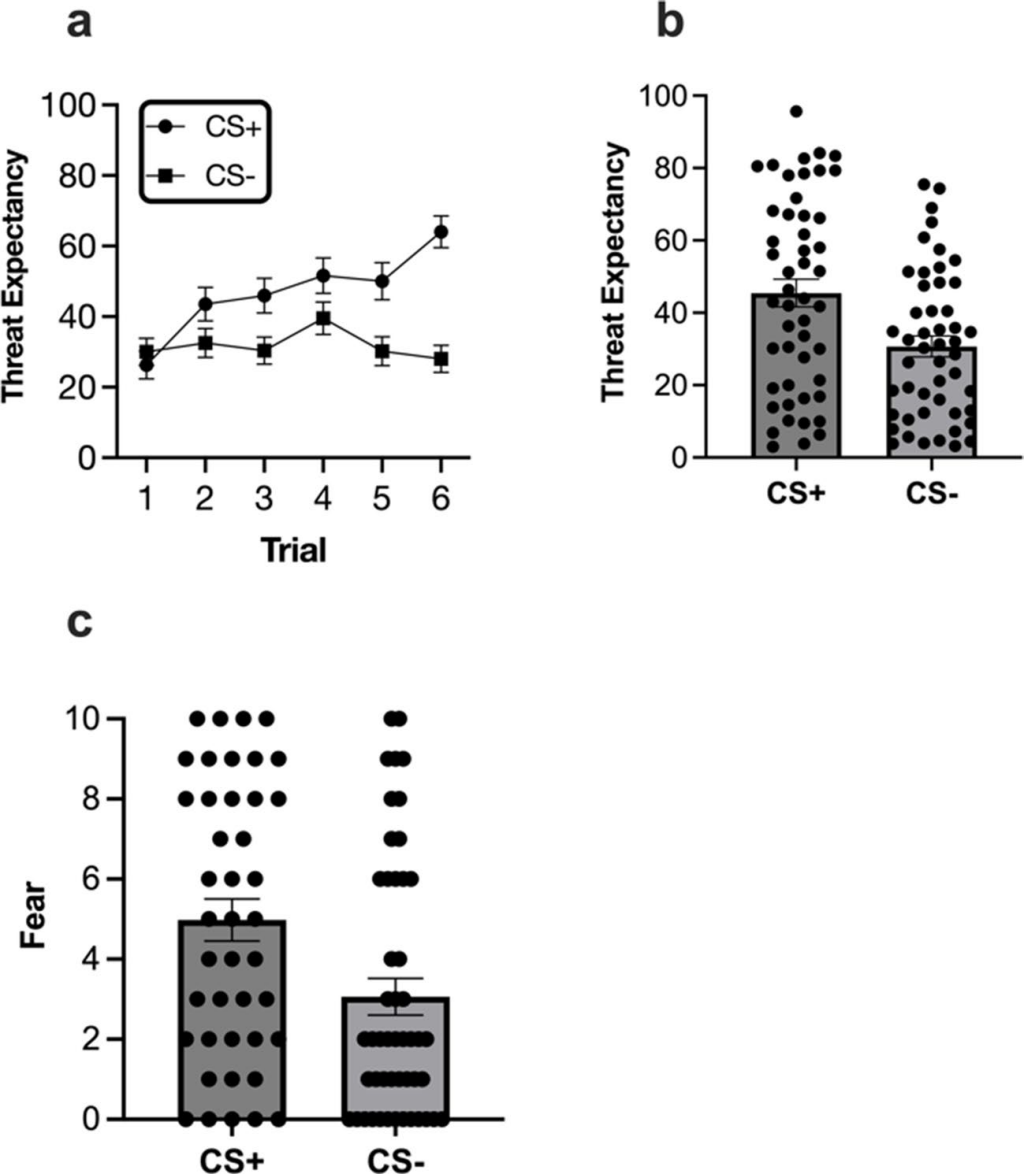
(**a**) Trial by trial, (**b**) grouped mean and individual values for threat expectancy and (**c**) grouped mean and individual values for fear ratings in fear conditioning. Error bars are SEM

During fear conditioning, main effects of stimuli and trial were superseded by a stimulus × trial interaction effect, *F* (3.60, 122.53) = 6.06, *p* < .001, η_p_^2^ = .15, 95% CI [.05, .24], BF_10_ > 100. Simple main effects revealed threat expectancy was comparable at the start of conditioning (Trials 1, 2 and 4, smallest *p* = .06), but was higher for CS+ at the end (Trials 3, 5 and 6, largest *p* < .001), thus indicating successful differential conditioning (Fig. 2).

Differential fear conditioning was also evident in fear ratings with CS+ (*M* = 5.09; *SD* = 3.44) receiving higher fear ratings than CS- (*M* = 3.00; *SD* = 3.20), *t* (42) = - 2.74, *p* = .009, *d* = .42, 95% CI [.10, .73], BF_10_ = 4.33.

### Generalisation

**Fig. 3:**
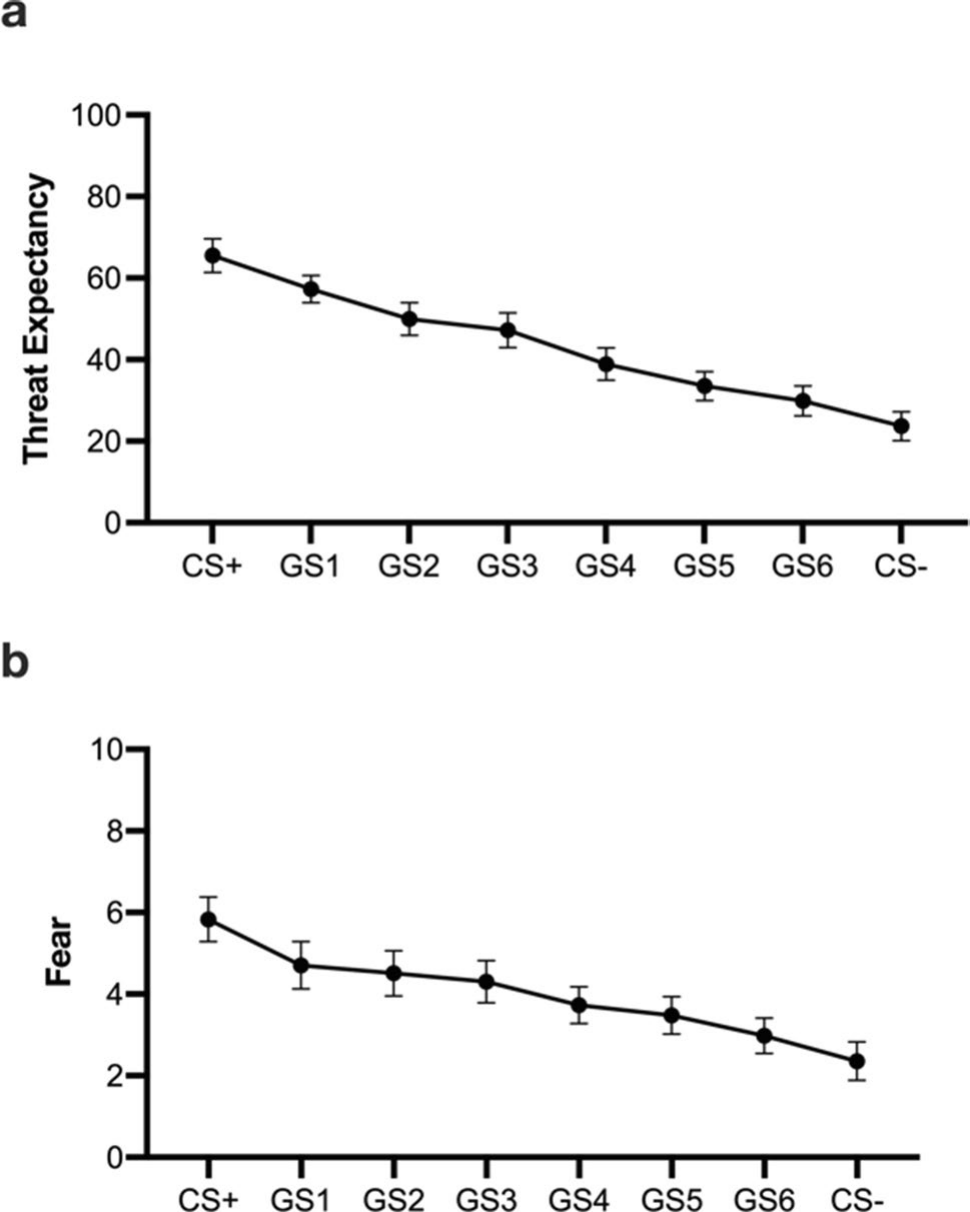
Mean threat expectancy (**a**) and fear ratings (**b**) averaged per four-trials, for all stimuli during the crucial generalisation test phase. Error bars are SEM

Trial by trial threat expectancy ratings during generalisation testing were averaged per four trials for each stimulus (CS+, GS1-6, CS-). A repeated-measures ANOVA revealed a main effect of threat expectancy ratings for the factor stimulus (CS+, CS-, GS1-6), *F* (2.65, 129.76) = 25.12, *p* < .001, η_p_^2^ = .34, 95% CI [.25, .41], BF_10_ > 100, with a negatively linear decrease in ratings across the spectrum of CS+, GS1 – GS6 and CS-, *t* (49) = - 6.86, *p* < .001 (see Fig. 3).

Post hoc-corrected t-tests revealed that CS+ was rated higher than all other stimuli (smallest *t* = 3.33, *p* = .03, *d* = .53, 95% CI [.00, 1.07]) except for GS+1. GS1 ratings were comparable to GS2 and GS3 ratings but higher than ratings for GS4-6 and CS- (smallest *t* = 5.06, *p* < .001, *d* = .81, 95% CI [.24, 1.38]). GS2 ratings were comparable to GS3 ratings but higher than ratings for GS4-6 and CS- (smallest *t* = 3.32, *p* = .03, *d* = .53, 95% CI [.00, 1.07]). GS3 ratings did not differ from GS4 ratings but were higher than ratings for GS5-6 and CS- (smallest *t* = 3.57, *p* = .01, *d* = .57, 95% CI [.03, 1.11]). GS4 ratings were comparable to GS5-6 ratings but higher than ratings for CS- (*t* = 3.30, *p* = .03, *d* = .53, 95% CI [.00, 1.06]). GS5, GS6 and CS- ratings were comparable.

Fear ratings also differed during generalisation testing with a main effect for the factor stimulus (CS+, CS-, GS1-6), *F* (2.86, 125.73) = 11.16, *p* < .001, η_p_^2^ = .20, 95% CI [.08, .32], BF_10_ > 100 and a negatively linear decrease in ratings across the spectrum of CS+, GS1 – GS6 and CS-, *t*(44) = - 4.56, *p* < .001 (see Fig. 3). Post hoc-corrected t-tests revealed that CS+ was rated comparably to GS1–3 but higher than GS4–6 and CS- (smallest *t* = 3.99, *p* = .002,* d* = .61, 95% CI [.08, 1.14]). GS1 was rated comparably to GS2–4, but higher than GS5–6 and CS- (smallest *t* = 3.19, *p* = .04,* d* = .49, 95% CI [-.03, 1.00]). GS2 was rated comparably to GS3–6, but higher than CS- (*t* = 4.62, *p* < .001, *d* = .69, 95% CI [.16, 1.25]). GS3 was rated comparably to GS4–6, but higher than CS- (*t* = 4.54, *p* < .001, *d* = .68, 95% CI [.15, 1.24]). GS4 was rated comparably to GS5–6, but higher than CS- (*t* = 3.40, *p* = .02, *d* = .51, 95% CI [.00, 1.04]). GS5, GS6 and CS- were rated comparably.

### Predictors of generalisation

Table 1 shows the descriptive statistics and Pearson correlation coefficients for each of the questionnaire measures and participants’ average threat expectancy and fear ratings for the generalisation stimuli (i.e., GS1-6).

**Table 1:** Mean scores (standard deviations) for each of the questionnaires, generalised threat expectancy and generalised fear and the Pearson correlation coefficients between these measures

Scale	Mean (SD)	1	2	3	4	5	6	7
*COVID-19 Fear (1)*	55.40 (10.27)	-	.36*	.25	.40**	.52***	.30*	.44**
*Worry (PSWQ) (2)*	45.54 (8.08)		-	.53***	.66***	.60***	.32*	.32*
*Depression (PHQ-9) (3)*	7.12 (6.31)			-	.72***	.49***	.00	.09
*Anxiety (GAD-7) (4)*	7.00 (5.28)				-	.57***	.05	.16
*Intolerance of Uncertainty (IUS) (5)*	36.02 (9.95)					-	.15	.25
*Generalised threat expectancy (6)*	43.82 (21.47)						-	.62***
*Generalised fear (7)*	3.99 (2.80)							-

* denotes statistical significance < .05; ** denotes statistical significance < .01; *** denotes statistical significance <.001

A standard multiple regression (using the “forced entry” method; i.e., all predictors were entered into the model simultaneously) with the questionnaire measures as predictors and average threat ratings to GS1–6 as the outcome approached significance, *F* (5, 44) = 2.41, *p* = .051,* R*^2^ = .22, adjusted *R*^2^ = .13, Cohen’s *f*^2=^ .28, 95% CI [.04, .66], BF_10_ = .83. Worry positively predicted generalised threat expectancy, although the Bayes factor only provided anecdotal evidence (β = .49, *p* < .05, BF_10_ = 2.33). All other predictors were non-significant (*p*s > .05).

An identical regression with average fear ratings to GS1–6 as the outcome was significant, although the Bayes factor only provided anecdotal evidence, *F* (5, 43) = 2.78, *p* = .03, *R*^2^ = .24, adjusted *R*^2^ = .16, Cohen’s *f*^2=^ .31, 95% CI [.06, .72], BF_10_ = 1.46. Fear of COVID-19 positively predicted fear ratings to GS1–6 (β = .42, *p* < .01, BF_10_ = 21.23). All other predictors were non-significant (*p*s > .05).

## Discussion

The current study examined whether a generalisation gradient was evident in an online threat conditioning task using COVID-19 relevant stimuli (i.e., busy or quiet shopping street/mall scenes) during COVID-19 lockdown restrictions. As predicted, following fear conditioning with CS+ and CS-, we observed a generalisation gradient in threat value with both threat expectancy and fear ratings. That is, ratings decreased in a downward linear manner along the continuum of CS+, GS1–6 and CS-. We also found evidence that threat ratings to the generalisation stimuli (i.e., GS1–6) were positively predicted by worry (as measured by the PSWQ), while fear ratings were positively predicted by COVID-19 fear (as measured by the FCQ). Caution must be maintained though when interpreting these relationships given the inconclusive Bayes factors. These findings do, however, add to the generalisation literature by examining threat expectancy and fear using a remote online task that incorporated COVID-19 relevant stimuli and by demonstrating how clinically relevant measures of worry and COVID-19 fear may influence generalised threat expectancy and fear.

The findings observed in this study are largely consistent with previous literature. For instance, Lissek et al. (2008) also observed a generalisation gradient of fear when using psychophysiological measures such as eye-blink startle reflex, whilst Boddez et al. (2019) found generalisation gradients of art appreciation (based on participants’ evaluative ratings) after positive and negative information was provided about specific artworks. The findings of the current study also demonstrate the utility of remote online tasks in examining fear-learning phenomena such as generalisation, which is consistent with recent studies that have examined threat expectancy, fear and avoidance remotely online (e.g., Cameron et al., 2022; McGregor et al., 2021; Purves et al., 2019). The literature has been somewhat heterogenous, however, as to whether clinically relevant trait measures can be used to predict the degree of generalisation that participants’ display. Whilst some studies have identified that anxiety can influence stimulus generalisation (Haddad et al., 2012), other traits such as worry (Dunning & Hajcak, 2015) have been shown to have little to no influence on generalisation, which is inconsistent with our results. To our knowledge, however, no studies have until now explored the relationship between COVID-19 fear and generalised threat expectancy and fear. Further research should examine the impact of other trait variables on remote-based fear generalisation tasks, perhaps with larger samples and at different time-points since the pandemic.

The primary implications of these findings are threefold. Firstly, these findings demonstrate how threat expectancy and fear can generalise to environments based on crowd density. Throughout the pandemic, crowded public areas were associated with potential danger (due to physical proximity to others and the possibility of infectious transmission), thus it could be that fear continues to generalise to crowded areas for some individuals as we learn to live with COVID-19. This may therefore produce agoraphobic-like behaviour in those with high levels of COVID-19 fear, leading them to avoid public spaces (e.g., shopping malls, festivals, airports). Moreover, the finding revealing stronger differential conditioning (i.e., higher CS+/CS- differentiation) when the busier street scene served as the CS+ than when the quieter street scene was the CS+, may have relevance for theoretical debates concerning mechanisms underlying enhanced threat learning to specific classes of stimuli (Åhs et al., 2018; Öhman & Mineka, 2001; Stussi et al., 2015, 2018, 2019, 2021). The present findings contribute to these debates by demonstrating a likely degree of preparedness or affective relevance for the chosen COVID-19 street scenes. Indeed, these effects were likely due, at least in part, to the real-world salience of the CSs and GSs, especially as data collection occurred during the pandemic when such scenes were commonplace and potentially threatening. Secondly, the identification of possible clinically relevant predictors (i.e., worry and COVID-19 fear) of generalised threat expectancy and fear provides insight into potential therapeutic targets for those who are most likely to experience generalised threat expectancy and fear in relation to crowded areas Previous studies have identified that females, older populations, and members of the Black, Asian and Minority Ethnic community are likely to report higher COVID-19 fear (Niño et al., 2021; Reznik et al., 2021). As such, it is possible that these populations are more susceptible to greater generalised fear. Thirdly, the remote online nature of the task demonstrates the utility of online tasks for examining generalisation of fear remotely. The online task used here could be easily adapted to a range of contexts to examine baseline levels of generalised threat expectancy and fear and to assess the effectiveness of treatment interventions to reduce generalised fear such as discrimination training (Ginat-Frolich et al., 2017; Lommen et al., 2017).

Nevertheless, there are several limitations to the current study that are important to consider. Due to the remote online nature of the task, we were unable to collect psychophysiological measures, and to ensure that the US delivery (i.e., the aversive sound) was standardised for all participants. Whilst we did instruct participants to turn their volume up to full, participants could still manipulate the volume during the task. To address this, we did include checks at the beginning and the end of the task where participants were asked whether they could hear the scream (and all indicated that they could). It would be salutary to pre-test stimulus valence to mitigate potentially elevated threat value (Cameron et al., 2023). The female sample also limits the generalisability of the findings, particularly as females have been shown to report elevated levels of COVID-19 fear (Reznik et al., 2021). It is possible, for instance, that this enhanced the impact of COVID-19 fear as a predictor of generalised fear. However, the gender effect of COVID-19 fear has been inconsistent (Erbiçer et al., 2021) and it is still important to understand generalised threat expectancy and fear in females during and since the pandemic. Finally, our sample size also limits the certainty to which we can determine with confidence the predictive ability of our measures. This is pertinent given that the Bayes factors for worry and COVID-19 fear provided only anecdotal evidence for the alternative hypothesis (Lee & Wagenmakers, 2013). The partial support for the role of worry possibly reflects a generalised absence of stimulus specificity for the feared object (e.g., COVID-19 infection) in the self-report measures provided. To identify small-to-medium effects a larger sample would be needed. As such, future research should seek to examine generalisation with the same task stimuli and measures using a larger, diverse sample that would also allow examination of a range of clinical and socio-demographic predictors of generalisation.

In conclusion, the current study provides evidence of a generalisation gradient with measures of threat expectancy and fear when administering a remote online active threat conditioning task that used COVID-19 relevant stimuli. These findings support previous literature that has examined generalisation using lab-based procedures. The current study also shows that worry and COVID-19 fear, respectively, predict generalised threat expectancy and fear to images of busy/quiet streets based on crowd density. However, replication of these findings with larger sample sizes is needed given that the Bayes factors for the trait measures (i.e., worry and COVID-19 Fear) only provided anecdotal evidence in favour of their predictability. These findings add to the body of literature examining generalisation and hold value as we transition back to pre-pandemic activities in busy social environments.

## Supplementary Information

Below is the link to the electronic supplementary material.

Supplementary file1 (DOCX 393 KB)

## Data Availability

The datasets generated during and/or analysed during the current study are available from the OSF here.

## Footnotes

1 Given the global public health conditions under which data were collected, it was perhaps unsurprising that the busier street scene served as a more effective CS+ than CS- across conditioning phases (OSM). At the time, COVID-19 transmission was considered likely in busy shopping malls and streets, among crowds, and in other situations where social distancing was either difficult or impossible.
