# Supplementary material for: Far from the threatening crowd: Generalisation of conditioned threat expectancy and fear in COVID-19 lockdown
Source: Learn Behav. 2024 Jan 29;52(3):262–71. doi: 10.3758/s13420-024-00625-4 (PMC11408548; doi:10.3758/s13420-024-00625-4)
Supplement: Supplementary file 1 — Supplementary file1 (DOCX 393 KB) [file 13420_2024_625_MOESM1_ESM.docx]

**Supplementary materials**

**Generalization test phase with individual values.**


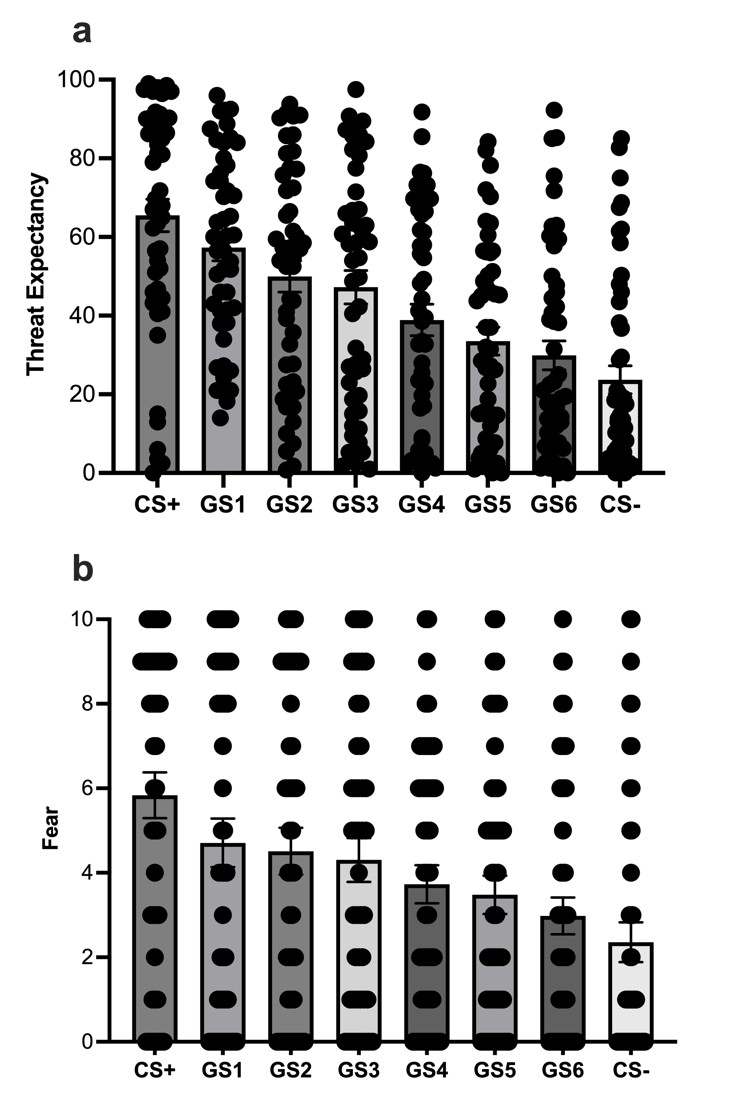


Figure S1 – Mean threat expectancy and mean fear ratings with individual values for all stimuli presented in the generalization test phase.

**Regression tables for multiple regression analyses reported in the manuscript:**

Generalized threat expectancy:

**Table 1** – *B*, SE, *β, T and P* of a multiple linear regression performed with COVID-19 Fear, GAD-7, PHQ-9, PSWQ and IUS as the predictors and *average threat ratings to GS1-GS6* as the outcome.

|  | *B* | *SE* | *β* | *T* | *P* |
| --- | --- | --- | --- | --- | --- |
| Intercept | -32.51 | 22.66 | -- | -1.44 | .16 |
| COVID-19 Fear | .63 | .33 | .30 | 1.90 | .06 |
| GAD-7 | -1.06 | .91 | -.26 | -1.16 | .25 |
| PHQ-9 | -.32 | .66 | -.10 | -.49 | .63 |
| PSWQ | 1.31 | .50 | .49 | 2.60 | .01 |
| IUS | -.24 | .41 | -.11 | -.59 | .56 |

Note. *B = unstandardised; SE* = Standard Error; *β* = standardised; T = t value; *P* = p value

Generalized fear expectancy:

**Table 2** – *B*, SE, *β, T and P* of a multiple linear regression performed with COVID-19 Fear, GAD-7, PHQ-9, PSWQ and IUS as the predictors and average fear ratings to GS1-GS6 as the outcome.

|  | *B* | *SE* | *β* | *T* | *P* |
| --- | --- | --- | --- | --- | --- |
| Intercept | -6.14 | 2.91 | -- | -2.11 | .041 |
| COVID-19 Fear | .12 | .04 | .42 | 2.69 | .01 |
| GAD-7 | -.08 | .12 | -.15 | -.70 | .49 |
| PHQ-9 | -.01 | .09 | -.03 | -.14 | .89 |
| PSWQ | .11 | .07 | .32 | 1.70 | .10 |
| IUS | -.02 | .05 | -.06 | -.32 | .75 |

Note. *B = unstandardised; SE* = Standard Error; *β* = standardised; T = t value; *P* = p value

**Sub-group analyses for each of the counterbalanced (CB) groups**:

There were two counterbalanced groups in the experiment. **In CB1 (n = 24) the busy street scene served as a CS+ and the quiet street scene served as CS-. In CB2 (n = 26) the quiet street scene served as a CS+ and the busy street scene served as CS-**. Given that COVID-19 transmission was most likely in busy shopping streets, crowds and other situations where social distancing was either difficult or impossible it was likely that busy street scene would serve as a more effective CS+ than the quiet street. As such, we explored this in analyses of each of the conditioning phases for each of the counterbalanced groups.

**Habituation:**

*Threat expectancy ratings*

Independent samples t-tests revealed that there was no significant difference in threat ratings of the CS+ between CB1 (M = 38.22; SD = 34.61) and CB2 (M = 20.56; SD = 23.37), *t* (23) = 1.53, *p* = .14, BF_10_ = .86. There was also no difference in threat ratings of the CS- between CB1 (M = 18.50; SD = 13.01) and CB2 (M = 25.81; SD = 21.04), *t*(32) = 1.23, *p* = .23, BF_10_ = .59.

*Fear ratings*

Mann-Whitney tests were conducted as the equal variance assumption was not met. There was a significant difference in fear ratings of the CS+ between CB1 (*M* = 4.05; *SD* = 3.07) and CB2 (*M* = 1.32; *SD* = 2.06), *W* = 292.00, *p* = .01, BF_10_ = 1.50. There was also a significant difference in fear ratings of the CS- between CB1 (M = 1.38; SD = 1.37) and CB2 (M = 3.20; SD = 2.90), *W* = 202.50, *p* = .05, BF_10_ = 1.32. These results reveal that the busier street scene elicited more fear at this stage than the quieter street scene in group CB1 and CB2.

**Fear conditioning:**

*Threat expectancy ratings*

A mixed model 2 × 2 × 6 ANOVA was conducted with the factors CB group (CB1 vs CB2), stimulus (CS- vs CS+) and Trial (1-6). There was a significant three-way interaction, *F* (3.74, 123.429) =3.94, *p* = .01, η_p_^2^ = .42, BF_10_ = 99.25. As such, we conducted separate follow up two-way repeated measures ANOVAs for each CB group with the factors stimulus (CS- vs CS+) and trial (1-6).

For CB1 there was a significant main effect of trial, *F* (3.18, 50.99) = 3.09, *p* = .03, η_p_^2^ = .04, BF_10_ > 1.33 and stimulus, *F* (1,16) = 18.59, *p* < .001, η_p_^2^ = .31, BF_10_ = 65.33 but no interaction *F* (5, 80) = .86, *p* = .48, η_p_^2^ = .01, BF_10_ = .13. The CS+ was rated higher than CS- across the course of fear conditioning. Post hoc t-tests, however, revealed that the CS+ was rated significantly higher on the final trial of training than the first trial, *t*(21) =3.53, *p* < .01. For CS-, however, there was no change, *t*(21) = .70, *p* = .50, demonstrating that the fear conditioning phase did result in increased threat ratings of CS+ but not CS-.

For CB2 there was no effect of stimulus *F* (1, 17) = .27, *p* = .61, η_p_^2^ = .00, BF_10_ = .35, but a significant main effect of trial *F* (3.28, 55.74) = 8.46, *p* < .001, η_p_^2^ = .08, BF_10_ = 18.99 and a significant interaction between stimulus and trial *F* (3.08, 52.39) = 9.70, *p* < .001, η_p_^2^ = .15, BF_10_ > 100. Simple main effects were conducted to explore the stimulus by trial interaction. On Trial 1 the CS+ was rated lower than the CS-, *F* (1) = 12.46, *p* = .003, however, by Trial 6, CS+ rated higher than CS-, *F* (1) = 18.69, *p* = < .001 (all other trials smallest *p* = .138). Thus, demonstrating that whilst CS- (in this case the busy street) initially produced high ratings from participants, this was reversed by the end of training, therefore demonstrating successful fear conditioning.

*Fear ratings*

A 2 × 2 mixed model ANOVA was conducted on the fear ratings following fear conditioning with the factors group (CB1 vs CB2) and stimulus (CS- vs CS+). There was no main effect of group, *F* (1, 41) = 2.36, *p* = .132, η_p_^2^ = .02, BF_10_ = .51, however, there was an effect of stimulus, *F* (1, 41) = 10.87, *p* = .002, η_p_^2^ = .11, BF_10_ = 12.19 and a stimulus × group interaction, *F* (1, 41) = 8.64, *p* = .01, η_p_^2^ = .08, BF_10_ = 18.27. Simple main effects revealed that fear ratings were higher for CS+ (i.e., the busier street scene) than CS- for CB1, *F* (1) = 18.63, *p* < .001, but not CB2, *F* (1) = .07, *p* = .80.

**Generalisation**:

*Threat expectancy ratings*

A 2 × 8 mixed model ANOVA was conducted on the threat ratings during generalisation with the factors group (CB1 vs CB2) and stimulus (CS+, GS1-6, CS-). There was a main effect of stimulus, *F* (2.34, 114.04) = 28.54, *p* < .001, η_p_^2^ = .23, BF_10_ > 100, group, *F* (1, 48) = 17.96, *p* < .001, η_p_^2^ = .09, BF_10_ > 100, and a stimulus × group interaction, *F* (2.38, 114.04) = 6.70, *p* < .001, η_p_^2^ = .05, BF_10_ > 100. Simple main effects revealed that there was an effect of stimulus for CB1, *F* (7) = 29.75, *p* < .001 and CB2, *F* (7) = 9.34, *p* < .001.

For group CB1, there was a quadratic, *t* (161) = - 3.82, *p* < .001 and negatively linear decrease in ratings across the CS+, GS1 – GS6 and CS-, *t* (161) = -13.67, *p* < .001. Post-hoc t-tests revealed that CS+ and GS1- GS3 were rated comparably. However, GS4-6 and CS- were rated lower than CS+ (smallest *p* = .002). GS1 was rated comparably to CS+ and GS2-3, but higher than GS4-6 and CS- (*ps* < .001). GS2 was rated equally to CS+ and GS1-3, but higher than GS4-6 and CS- (smallest *p* = .004). GS3 was rated similarly to CS+ and GS1-2 but higher than GS4-6 and CS- (smallest *p* = .01). GS4 was rated higher than GS6 and CS- (smallest *p* = .04), but similar to GS5. GS5 was rated higher than CS- (*p* = .002). GS6 was rated comparably to CS- (*p* = .74).

For group CB2, there was a quadratic, *t* (175) = 3.99, *p* < .001 and a negatively linear decrease in ratings across the CS+, GS1 – GS6 and CS- *t* (175) = -6.99, *p* < .001. Post-hoc t-tests revealed that CS+ was rather higher than all other stimuli except GS1 (smallest *p* < .001). GS1 was rated higher than GS4-6 and CS- (smallest *p* = .02). However, GS2-G6 and CS- were rated comparably.

*Fear ratings*

A 2 × 8 mixed model ANOVA was conducted on the fear ratings following generalisation with the factors group (CB1 vs CB2) and stimulus (CS+, GS1-6, CS-). There was a main effect of stimulus, *F* (3.17, 136.27) = 12.69, *p* < .001, η_p_^2^ = .09, BF_10_ > 100, group, *F* (1, 43) = 11.48, *p* < .01, η_p_^2^ = .10, BF_10_ = 22.12, and a stimulus × group interaction, *F* (3.17, 136.27) = 12.37, *p* < .001, η_p_^2^ = .09, BF_10_ > 100. Simple main effects revealed that there was an effect of stimulus for CB1, *F* (7) = 21.08, *p* < .001 and CB2, *F* (7) = 3.50, *p* < .01.

For group CB1, there was a quadratic, *t* (161) = - 3.08, *p* < .01 and negatively linear decrease in ratings across the CS+, GS1 – GS6 and CS-, *t* (161) = -11.62, *p* < .001. Post-hoc t-tests revealed that CS+, GS1- GS4 were rated comparably. However, GS5-6 and CS- were rated lower than CS+ (smallest *p* < .001). GS1 was rated higher than GS4-6 and CS- (smallest *p* < .02). GS2 was rated higher than GS5-6 and CS- (smallest *p* < .001). GS3 was rated higher than GS5-6 and CS- (smallest *p* < .001).GS4 was rated higher than GS6 and CS- (smallest *p* = .05). GS5 was rated higher than CS- (*p* = .04), whilst GS6 was rated comparably to CS- (*p* =. 99).

For group CB2, there was a quadratic, *t* (140) = 3.33, *p* < .01 and cubic effect *t* (140) = -3.21, *p* = .002, but no linear decrease in the ratings *t* (140) = -1.34, *p* = .18. Post-hoc t-tests revealed that CS+ was rather higher than all other stimuli (smallest *p* = .01). However, all other stimuli were rated comparably.


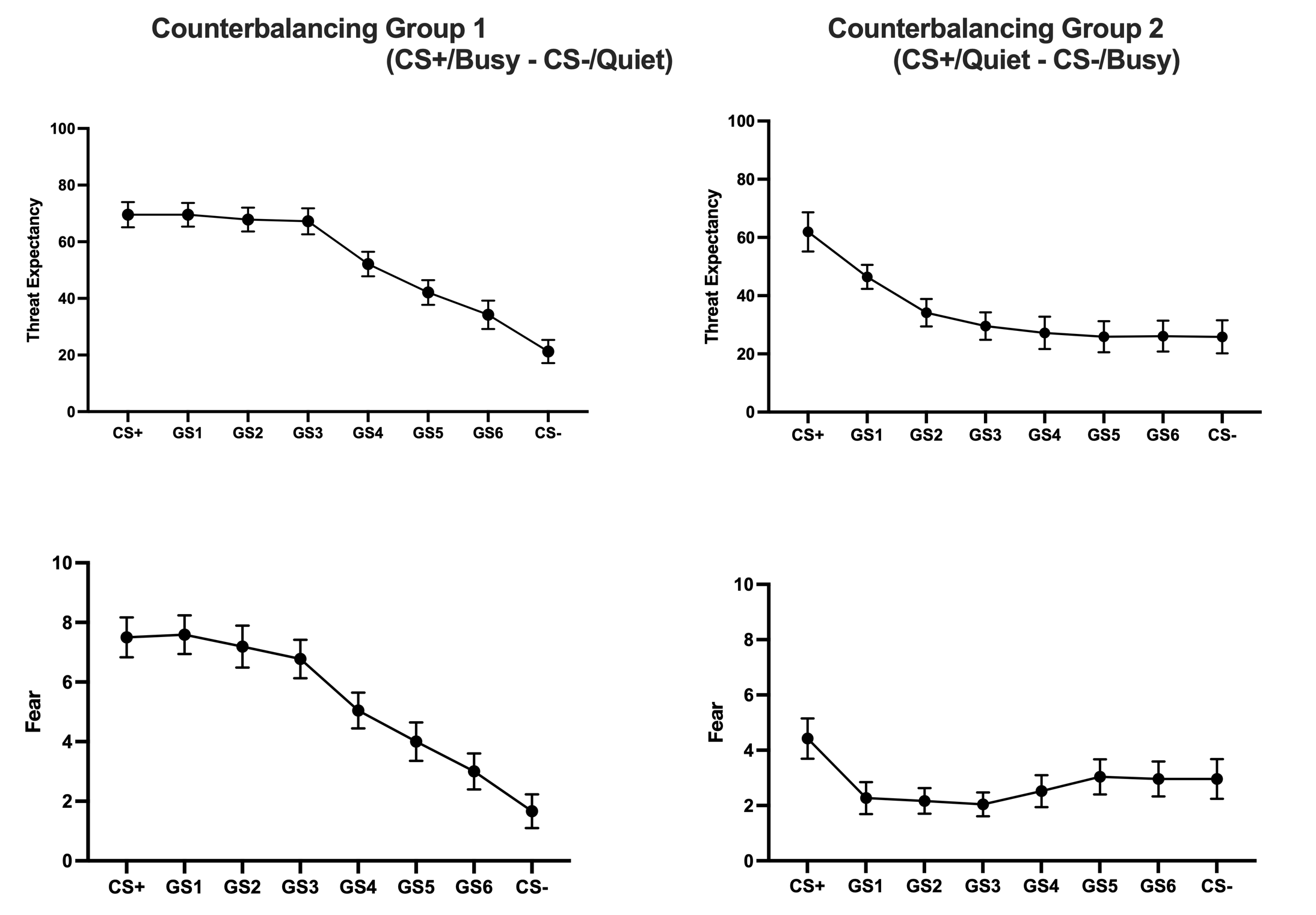


***Figure S2***: Mean threat expectancy and fear ratings during generalization testing for participants in counterbalancing group 1 (CB1) and counterbalancing group 2 (CB2). Error bars show SEM.
